# Supplementary material for: Causal Relationship between Aging and Anorexia Nervosa: A White-Matter-Microstructure-Mediated Mendelian Randomization Analysis
Source: Biomedicines. 2024 Aug 16;12(8):1874. doi: 10.3390/biomedicines12081874 (PMC11351342; doi:10.3390/biomedicines12081874)
Supplement: Supplementary file 1 [file biomedicines-12-01874-s001.zip › Supplementary Figure/Figure S8/Leaveoneout plot of MR analysis for TL to ubm-b-1878.pdf]

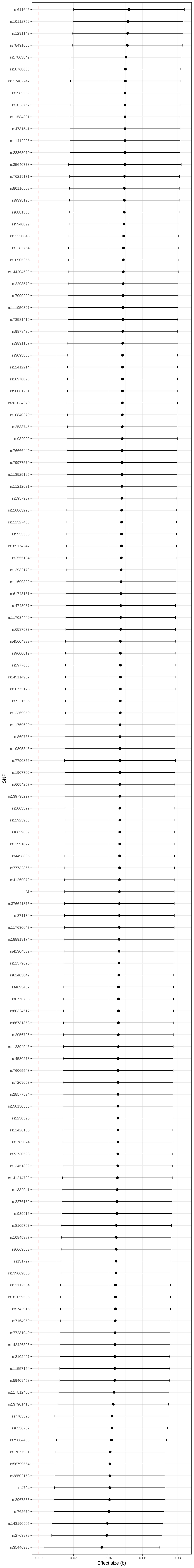

rs611646
rs10112752
rs1291143
rs78491606
rs17803849
rs10768683
rs117407747
rs1985369
rs1023767
rs11584821
rs4731541
rs11412296
rs28363070
rs35640778
rs76219171
rs80116508
rs9398196
rs6881568
rs9940099
rs13230646
rs2282764
rs10905255
rs144204502
rs2293579
rs7099229
rs111950327
rs73581419
rs9878436
rs3891167
rs3093888
rs12412214
rs16978028
rs56061761
rs202034370
rs10840270
rs2538745
rs932002
rs76666449
rs79977579
rs113525195
rs11212631
rs1957937
rs116863223
rs111527438
rs9955360
rs185174247
rs2555104
rs12932179
rs11699829
rs61748181
rs4743037
rs117034449
rs6587577
rs45604339
rs9600019
rs2977608
rs145114957
rs10773176
rs7221585
rs12369950
rs11769630
rs869785
rs10805346
rs7790856
rs1907702
rs6054257
rs139795227
rs1003322
rs12925933
rs6659669
rs11991877
rs4498805
rs77732866
rs41269079
A8
rs376641875
rs871134
rs117630647
rs188918174
rs41304832
rs11579626
rs61405024
rs4695407
rs6776756
rs80324517
rs66731853
rs2056726
rs112394943
rs4530278
rs76065543
rs7209057
rs28577594
rs150150565
rs2230590
rs11426156
rs3785074
rs73730598
rs12451892
rs141214782
rs1332941
rs2276182
rs939916
rs8105767
rs10845387
rs6669563
rs131797
rs139669835
rs11117354
rs182059286
rs5742915
rs7164950
rs77231040
rs142426306
rs8102497
rs11557154
rs59409453
rs117512405
rs137901416
rs7705526
rs6536702
rs75664430
rs17677991
rs56799554
rs28502153
rs4724
rs2967355
rs762679
rs143190905
rs2763979
rs35446936
SNP
0.00
0.02
0.04
0.06
0.08
Effect size (b)
